# RGB Indices Can Be Used to Estimate NDVI, PRI, and Fv/Fm in Wheat and Pea Plants Under Soil Drought and Salinization

**DOI:** 10.3390/plants14091284

**Published:** 2025-04-23

**Authors:** Yuriy Zolin, Alyona Popova, Lyubov Yudina, Kseniya Grebneva, Karina Abasheva, Vladimir Sukhov, Ekaterina Sukhova

**Affiliations:** Department of Biophysics, N.I. Lobachevsky State University of Nizhny Novgorod, 603950 Nizhny Novgorod, Russia; uchebnayap.zolin@gmail.com (Y.Z.); silverkumiho@mail.ru (A.P.); lyubovsurova@mail.ru (L.Y.); grebneva.kseniya01@mail.ru (K.G.); karinarutter@yandex.ru (K.A.); vssuh@mail.ru (V.S.)

**Keywords:** soil drought, salinization, RGB indices, ExG, VARY, NDVI, PRI, Fv/Fm, pea, wheat

## Abstract

Soil drought and salinization are key abiotic stressors for agricultural plants; the development of methods of their early detection is an important applied task. Measurement of red-green-blue (RGB) indices, which are calculated on basis of color images, is a simple method of proximal and remote sensing of plant health under the action of stressors. Potentially, RGB indices can be used to estimate narrow-band reflectance indices and/or photosynthetic parameters in plants. Analysis of this problem was the main task of the current work. We investigated relationships of six RGB indices (r, g, b, ExG, VEG, and VARI) to widely used narrow-band reflectance indices (the normalized difference vegetation index, NDVI, and photochemical reflectance index, PRI) and the potential quantum yield of photosystem II (Fv/Fm) in wheat and pea plants under soil drought and salinization. It was shown that investigated RGB indices, NDVI, PRI, and Fv/Fm were significantly changed under the action of both stressors; changes in some RGB indices (e.g., ExG) were initiated on the early stage of action of drought or salinization. Correlation analysis showed that RGB indices (especially, ExG, VARY, and g) were strongly related to the NDVI, PRI, and Fv/Fm; linear regressions between these values were calculated. It means that RGB indices measured by simple and low-cost color cameras can be used to estimate plant parameters (NDVI, PRI, and Fv/Fm) requiring sophisticated equipment to measure.

## 1. Introduction

The climatic changes, which are accompanied by increasing temperature and irregular precipitations, and faulty irrigation contribute to soil drought and salinization [1,2]. It is known that drought and salinization decrease water exchange suppressing the photosynthetic CO_2_ assimilation through stomata closure [2,3] and disrupt transport of nutrients [3,4]. The oxidative stress, which can also be induced by action of these stressors, causes the destruction of biological membranes and damage of photosynthetic machinery [2,5]. As a result, the prolonged action of soil drought and salinization strongly decreases plant growth and productivity [1,2]. It means that early detection of the action of drought and salinization on plants is an important task for agriculture. Solution of this task can be based on remote and proximal sensing of characteristics of plants using optical methods which are a powerful tool of plant cultivation [6,7].

Light plays an important role in plant life because it is an energy source for photosynthesis and productivity [8] and a regulator of physiological processes [9], water exchange [10], development, and growth [11]. On the other hand, changes in absorption and reflectance of light can show information about processes in plants [12,13]; analysis of reflected light (back scattered light) is especially important for remote and proximal sensing of plants. Particularly, it is known that a reduction in pigment content (or its photoinhibition), which can be induced by the action of adverse factors (including drought and salinization), decreases light absorption and increases reflectance in plants; on the other hand, structural changes during plant development or under action of stressors influence the scattering of light in the leaf lamina [12,13,14,15,16]. Considering an influence of mineral nutrition on accumulation of photosynthetic pigments and development of leaves [15,17], characteristics of this nutrition can also be estimated on the basis of plant optical characteristics. As a result, the remote and proximal sensing of plants can be the basis of estimation of their growth, prediction of crops, effective application of fertilizers, detection of influence of stressors, and others [7,18,19]. Thus, development of methods of increasing informativity of analysis of images, which can be the basis of decision making, is an important problem of plant cultivation.

Spectral imaging is based on measurement, analysis, and interpretation of spectral characteristics of reflected light which is back scattered by internal structures in leaf lamina. There are three main approaches for imaging of reflected light [12,13,20]: imaging of reflectance spectra (the hyperspectral imaging), imaging of reflectance in several narrow spectral bands (the multispectral imaging), and imaging of reflectance in three broad spectral bands (the red (R)-green (G)-blue (B) imaging).

Analysis of results of the hyperspectral and multispectral imaging can be based on calculation of narrow-band reflectance indices [12,15,21], which are often calculated using reflectance in two or three narrow spectral bands and are non-dimensional. Particularly, the normalized difference vegetation index (NDVI) and photochemical reflectance index (PRI) are narrow-band reflectance indices, which are widely used in remote and proximal sensing of plants [16,20,22,23]; varying platforms (from handheld devices to unmanned aerial vehicles, airplanes, and satellites [6,7,12,16]) can be used for their measurements. It is known that the NDVI mainly shows total photosynthetic biomass and chlorophyll content in plants [12,24,25]. This reflectance index can be used to remote sensing of plant characteristics in various time ranges including multi-year time series [26,27,28]; in the last case, the NDVI can be used as the basis of calculation of a gross primary production of plants [26,27]. In contrast, PRI can also be related to fast changes in photosynthetic processes under action of adverse factors (e.g., excess light) through sensitivity of this index to transitions in the xanthophyll cycle [16,23,29] and to the shrinkage of chloroplasts [16,30]; both processes are stimulated by acidification of the chloroplast lumen and, therefore, are related to photosynthetic processes in plants (e.g., to the energy-depended component of the nonphotochemical quenching of chlorophyll fluorescence). Particularly, it is known that the NDVI and PRI are sensitive to drought and salinization and can be used to detect presence of these stressors [16,31].

Despite the efficiency of the hyperspectral and multispectral imaging for remote and proximal sensing of plants, these approaches require sophisticated and high-cost equipment [13], which restricts using the NDVI and PRI for plant monitoring. In contrast, RGB indices, which are calculated on basis of broad reflectance bands (R, G, and B) measured by color cameras, can be a simple and low-cost method of plant remote and proximal sensing [13]. It is known that RGB indices are strongly correlated with many plant characteristics, including concentration of chlorophylls and carotenoids [32,33], nitrogen content [19], biomass [34], leaf area index [35,36], and others. However, these relations can be dependent on many factors including the stage of development [35,37], plant cultivar and species [38,39], leaf thickness and overlapping [38,40], or conditions of measurement [13]. It means that development of methods to improve the efficiency of using RGB indices is an important task of plant remote and proximal sensing.

Searching RGB indices, which are strongly related to narrow-band reflectance indices (particularly, the NDVI and PRI) and can be used for their calculation (e.g., on the basis of regressions), is an important step in this development. The dependence of RGB indices [13] and the most of narrow-band reflectance indices including NDVI [24,25] and PRI [16] on content of photosynthetic pigments theoretically shows that RGB indices can be potentially used to estimate many narrow-band indices. There are numerous works which show the relationships of narrow-band reflectance indices to different plant characteristics and provide regression equations to describe these relationships (see, e.g., our review [12]). It is probable that analogues of narrow-band indices, which are calculated on basis of RGB indices, can also be used in these regressions providing a simple alternative of multispectral and hyperspectral imaging.

There are the few works showing correlations of RGB indices with narrow-band [41] and broad-band [33] reflectance indices; additionally, some works [42,43,44] show the reconstruction of hyperspectral images on the basis of RGB images using machine learning methods. These results show that RGB indices can be potentially used to estimate narrow-band reflectance indices; however, this problem requires further experimental investigations.

An alternative way of improving the efficiency of using RGB indices can be based on further investigations of relations between these indices and plant characteristics. Searching RGB indices, which are sensitive to photosynthetic parameters, can be especially important for remote and proximal sensing of plants. Particularly, RGB indices-based estimation of the potential quantum yield of photosystem II (Fv/Fm) under action of the stressors can be used as an important tool of monitoring of plant health because Fv/Fm is a sensitive indicator of plant photosynthetic performance, which is decreased under action of adverse factors and following photodamage of the photosynthetic machinery [45]. It is known that Fv/Fm can be strongly decreased under the action of drought [46,47] and salinization [48,49] in plants including pea and wheat [47,50,51].

Thus, our investigation was devoted to searching RGB indices strongly related to the NDVI, PRI, and Fv/Fm in pea and wheat plants under action of soil drought and salinization. Developing regression equations, which could be used to estimate the NDVI, PRI, and Fv/Fm on the basis of the detected RGB indices under the action of these stressors, was the second task of the current investigation. It was expected that the solution of both tasks could be the basis of tools to estimate narrow-band reflectance indices (NDVI, PRI) and photosynthetic parameters (Fv/Fm) under the action of drought and salinization using RGB images measured by simple and low-cost color cameras.

To solve these tasks, we analyzed six widely used RGB indices including the normalized red coordinate (r), normalized green coordinate (g), normalized blue coordinate (b), excess green index (ExG), vegetative index (VEG), and visible atmospherically resistance index (VARI) [13]. It was known that these RGB indices could be related to content of chlorophylls [13]; i.e., their relationships to photosynthetic parameters and narrow-band reflectance indices (at least, to indices, which were dependent on concentration of photosynthetic pigments) seemed to be probable.

## 2. Results

### 2.1. Influence of Soil Drought on RGB Indices, NDVI, PRI, and Fv/Fm

**Figure 1:**
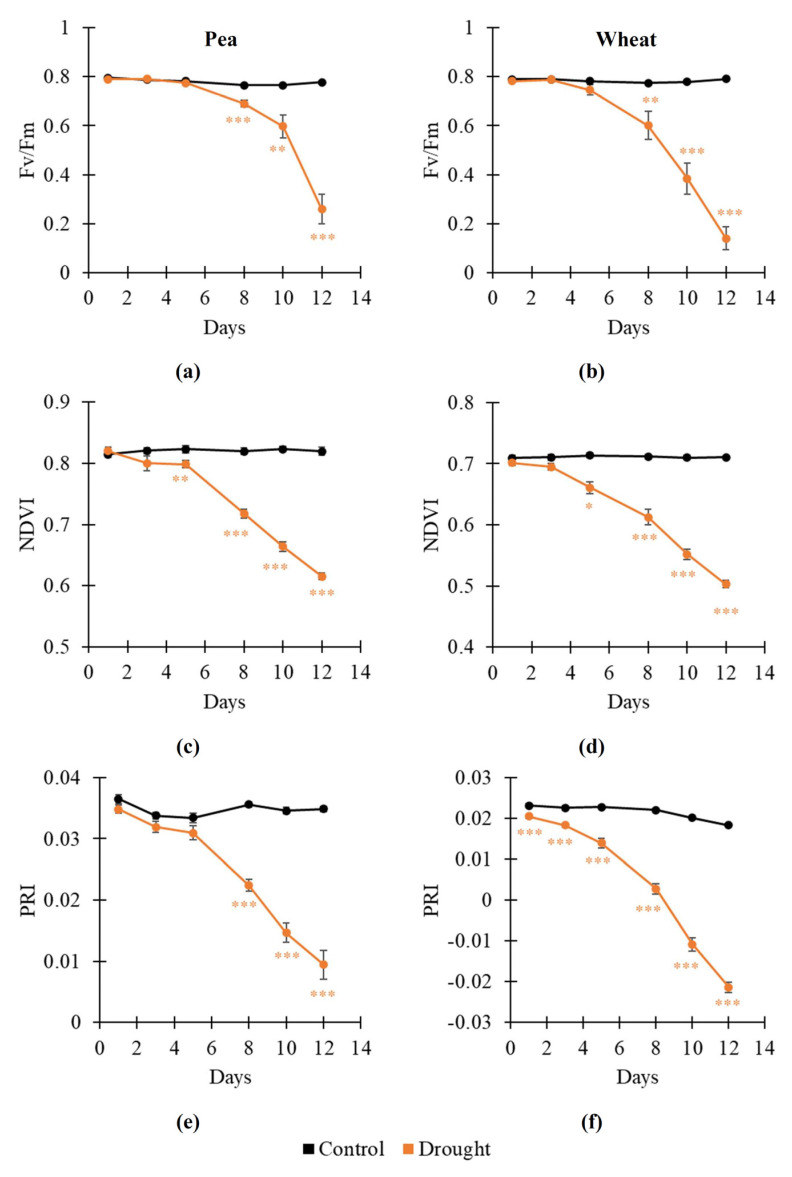
Dynamics of drought-induced changes in the potential quantum yield of the photosystem II (Fv/Fm) in pea (**a**) and wheat (**b**), the normalized difference vegetation index (NDVI) in pea (**c**) and wheat (**d**), and photochemical reflectance index (PRI) in pea (**e**) and wheat (**f**) (*n* = 10). Asterisks show significant differences between the plants under drought and control conditions (*, *p* < 0.05; **, *p* < 0.01; ***, *p* < 0.001).

The influence of soil drought on RGB indices, NDVI, PRI, and Fv/Fm in pea and wheat plants was analyzed on the first stage of the current investigation (Figure 1). It was shown (Figure 1a,b) that soil drought decreased Fv/Fm from about 0.8 to about 0.2 (pea plants) or about 0.1 (wheat plants) after 12 days of its action. A significant decrease was initiated from the 8th day of the drought action for both plants.

Analysis of narrow-band reflectance indices (NDVI and PRI) showed qualitatively similar results: both indices were decreased under the action of soil drought (Figure 1c–f). A significant decrease in the NDVI was initiated from the 5th day of the drought action in both pea and wheat plants. A significant decrease in the PRI was initiated from the 8th day of the drought action in pea plants; in contrast, it was initiated from the 1st day of soil drought in wheat plants. These results showed that the NDVI (in pea and wheat plants) and PRI (in wheat plants) could be more sensitive to drought action than Fv/Fm; this result supported the possibility of their using to detect the drought action on plants.

**Figure 2:**
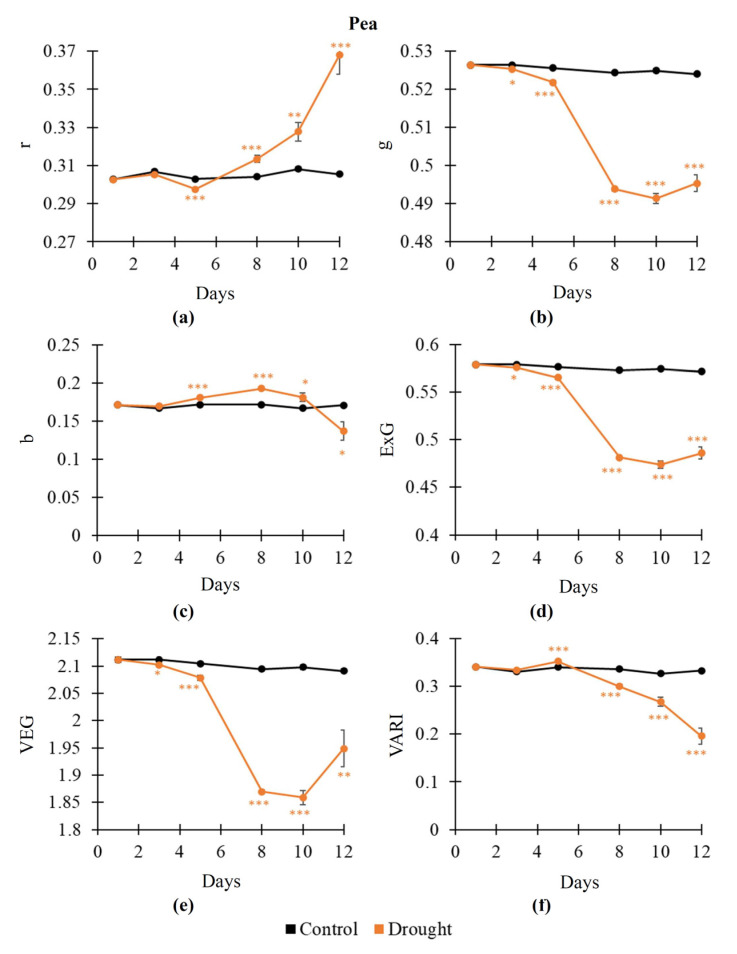
Dynamics of drought-induced changes in the normalized red coordinate (r) (**a**), normalized green coordinate (g) (**b**), normalized blue coordinate (b) (**c**), excess green index (ExG) (**d**), vegetative index (VEG) (**e**), and visible atmospherically resistance index (VARI) (**f**) in pea plants (*n* = 10). Asterisks show significant differences between the plants under drought and control conditions (*, *p* < 0.05; **, *p* < 0.01; ***, *p* < 0.001).

The influence of soil drought on RGB indices was further analyzed. It was shown (Figure 2) that changes in RGB indices in pea plants could have intricate dynamics. Particularly, the dynamic of drought-induced changes in r included significant decrease and following increase (Figure 2a); in contrast, dynamics of changes in b (Figure 2c) and VARI (Figure 2f) included significant increase and following decrease. g, ExG, and VEG were decreased under the drought action in pea plants (Figure 2b,d,e); significant decrease was initiated from the 3rd day of soil drought.

**Figure 3:**
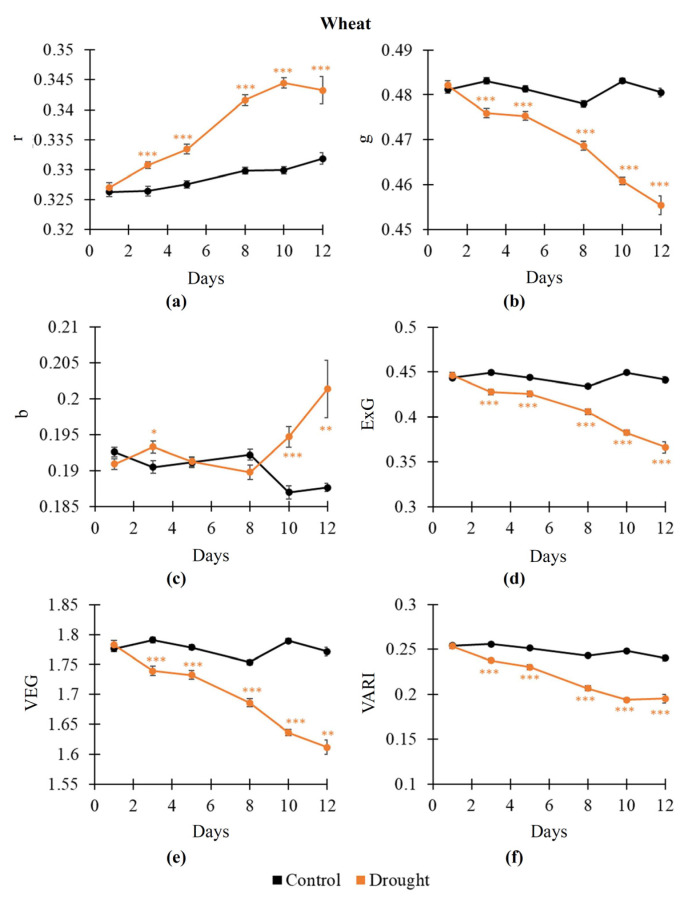
Dynamics of drought-induced changes in the normalized red coordinate (r) (**a**), normalized green coordinate (g) (**b**), normalized blue coordinate (b) (**c**), excess green index (ExG) (**d**), vegetative index (VEG) (**e**), and visible atmospherically resistance index (VARI) (**f**) in wheat plants (*n* = 10). Asterisks show significant differences between the plants under drought and control conditions (*, *p* < 0.05; **, *p* < 0.01; ***, *p* < 0.001).

Drought-induced changes in RGB indices in wheat plants had more simple dynamics (Figure 3). It was shown that r was significantly increased (Figure 3a); in contrast, g (Figure 3b), ExG (Figure 3d), VEG (Figure 3e), and VARI (Figure 3f) were significantly decreased under soil drought. Changes in the RGB indices (r, g, ExG, VEG, and VARI) were initiated from the 3rd day of the drought action; i.e., these indices were more sensitive to this action than the NDVI. In contrast, b was stably and significantly increased from the 10th day of the drought action (Figure 3c).

Thus, our results showed that the investigated RGB indices were mainly sensitive to action of soil drought; moreover, their changes could be initiated earlier than changes in Fv/Fm or narrow-band reflectance indices.

### 2.2. Influence of Salinization on RGB Indices, NDVI, PRI, and Fv/Fm

**Figure 4:**
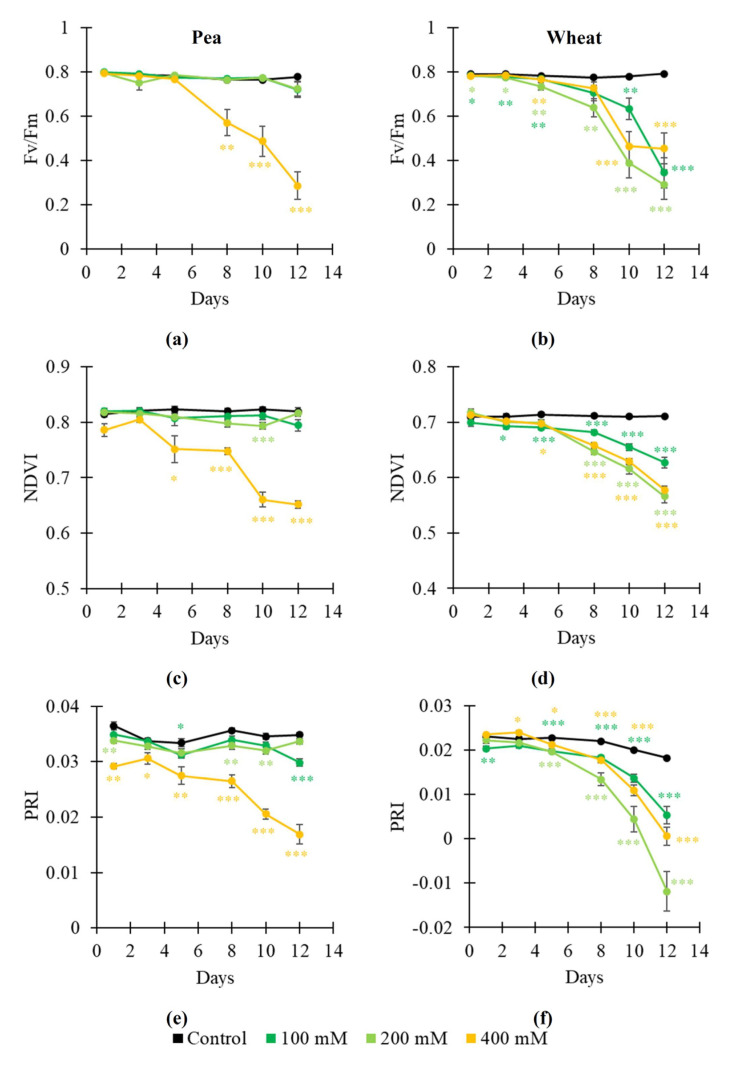
Dynamics of salinization-induced changes in the potential quantum yield of the photosystem II (Fv/Fm) in pea (**a**) and wheat (**b**), normalized difference vegetation index (NDVI) in pea (**c**) and wheat (**d**), and photochemical reflectance index (PRI) in pea (**e**) and wheat (**f**) (*n* = 10). The 100 mM, 200 mM, and 400 mM NaCl solutions were used to induce salinization. Asterisks show significant differences between the plants under drought and control conditions (*, *p* < 0.05; **, *p* < 0.01; ***, *p* < 0.001); the color of the asterisks shows the NaCl concentration.

An influence of salinization on RGB indices, NDVI, PRI, and Fv/Fm in pea and wheat plants was analyzed on the second stage of the current investigation (Figure 4). It was shown (Figure 4a) that the 400 mM NaCl treatment decreased Fv/Fm from about 0.8 to about 0.25 in pea plants after 12 days of this treatment; in contrast, the 100 mM and 200 mM NaCl treatments did not stably influence Fv/Fm. A decrease in Fv/Fm was initiated in pea plants from the 8th day of the 400 mM treatment. In wheat plants, all variants of the NaCl treatments (100, 200, and 400 mM) significantly decreased Fv/Fm from about 0.8 to about 0.3–0.5 after 12 days of these treatments (Figure 4b). A decrease in Fv/Fm was initiated in wheat plants from the 1st day of the 100 and 200 mM NaCl treatments and from the 5th day of the 400 mM NaCl treatment.

The influence of salinization on the NDVI was qualitatively similar. The 400 mM NaCl treatment decreased the NDVI in pea plants from the 5th day of this treatment (Figure 4c); in contrast, the 100 and 200 mM NaCl treatments weakly influenced the NDVI. In wheat plants, all salinization treatments decreased the NDVI from the 3rd (100 mM NaCl), 8th (200 mM NaCl), and 5th (400 mM NaCl) days of these treatments (Figure 4d). The NaCl treatments also decreased the PRI in pea and wheat plants. Decreasing PRI was initiated in pea plants from the 12th, 8th, and 1st days after treatments by 100, 200, and 400 mM NaCl, respectively (Figure 4e). In wheat plants, significantly decreasing the PRI was initiated from the 1st, 5th, and 3rd days after treatments by 100, 200, and 400 mM NaCl, respectively (Figure 4f).

**Figure 5:**
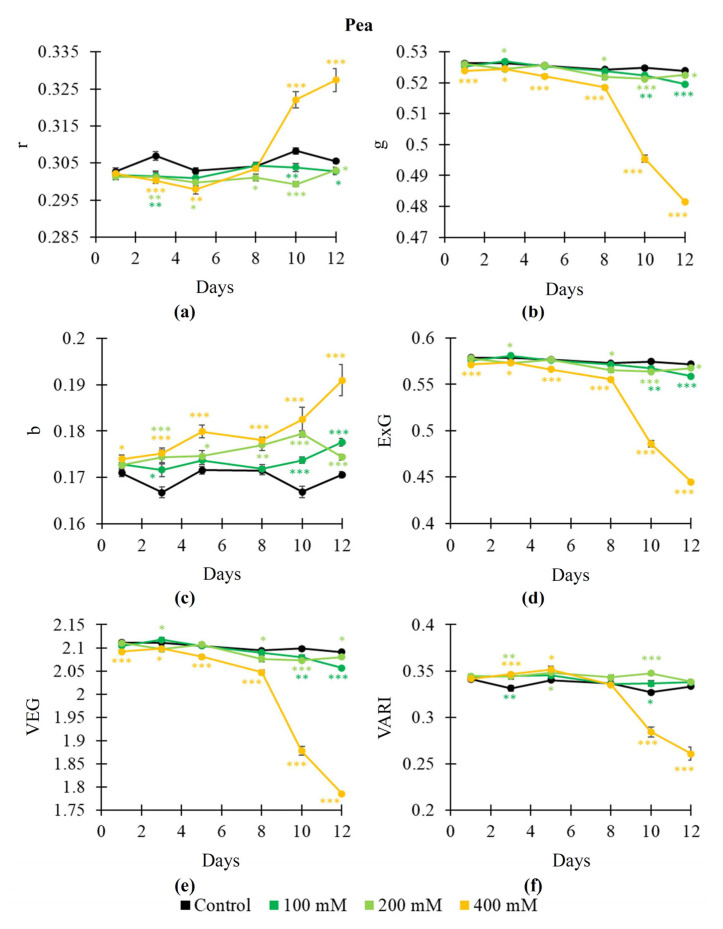
Dynamics of salinization-induced changes in the normalized red coordinate (r) (**a**), normalized green coordinate (g) (**b**), normalized blue coordinate (b) (**c**), excess green index (ExG) (**d**), vegetative index (VEG) (**e**), and visible atmospherically resistance index (VARI) (**f**) in pea plants (*n* = 10). The 100 mM, 200 mM, and 400 mM NaCl solutions were used to induce salinization. Asterisks show significant differences between the plants under drought and control conditions (*, *p* < 0.05; **, *p* < 0.01; ***, *p* < 0.001); color of the asterisks shows the NaCl concentration.

**Figure 6:**
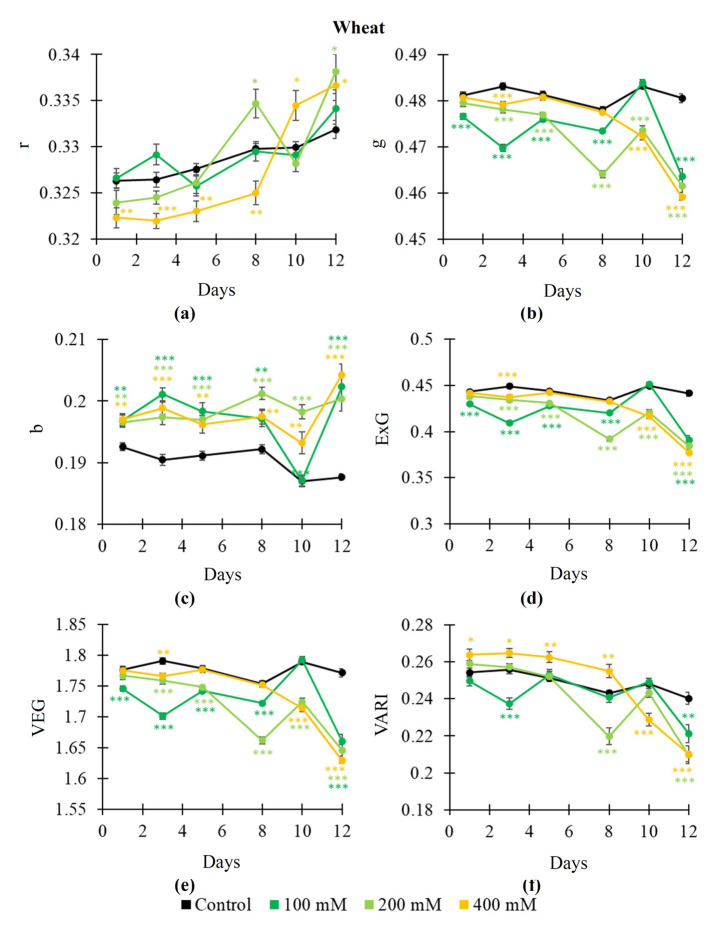
Dynamics of salinization-induced changes in the normalized red coordinate (r) (**a**), normalized green coordinate (g) (**b**), normalized blue coordinate (b) (**c**), excess green index (ExG) (**d**), vegetative index (VEG) (**e**), and visible atmospherically resistance index (VARI) (**f**) in wheat plants (*n* = 10). The 100 mM, 200 mM, and 400 mM NaCl solutions were used to induce salinization. Asterisks show significant differences between the plants under drought and control conditions (*, *p* < 0.05; **, *p* < 0.01; ***, *p* < 0.001); color of the asterisks shows the NaCl concentration.

The dynamics of salinization-induced changes in RGB indices were not stable (Figure 5 and Figure 6), especially, in wheat plants. Two-phasic changes could be observed for some indices (e.g., r and VARI in pea plants, Figure 5a,f). It was shown that salinization mainly increased r (Figure 5a) and b (Figure 5c) and decreased g (Figure 5b), ExG (Figure 5d), VEG (Figure 5e), and VARI (Figure 5f). The 400 mM NaCl treatment induced changes in indices with largest magnitudes; changes in some RGB indices (particularly, g, ExG, and VEG) were initiated from the 1st day of this treatment. Changes induced by the 100 and 200 mM NaCl treatments were not stable.

In wheat plants, salinization decreased g (Figure 6b), ExG (Figure 6d), and VEG (Figure 6e); in contrast, b was increased (Figure 6c). The salinization-induced changes in r (Figure 5a) and VARI (Figure 5f) were intricated: the 400 mM NaCl treatment decreased r and increased VARI from the 1st to 8th days and increased r and decreased VARI from 10th day. In contrast, the 100 and 200 mM NaCl treatments significantly influenced r and VARI in some time points only. It should be noted that experimental and control dynamics of changes in these RGB indices were mainly not stable; there were time points with insignificant differences with control values (e.g., the 5th and 8th days for changes in g), which were observed after points with significant differences (e.g., the 3rd day for changes in g).

Thus, our results showed that salinization-induced changes in Fv/Fm, NDVI, PRI, and, especially, RGB indices were more intricated than drought-induced changes in these parameters. However, some RGB indices were strongly sensitive to the salinization action; e.g., b was mainly increased after action of all the used NaCl treatments (Figure 5c and Figure 6c). Increasing b could be initiated from the 1st day of the NaCl treatment.

### 2.3. Relationships of RGB Indices to Fv/Fm, NDVI and PRI

Further, we analyzed the relationships of RGB indices to Fv/Fm, the NDVI, and PRI (Table 1). Pearson correlation coefficients were calculated for plants under control and drought conditions or under control and salinization conditions. We did not analyze control plants only or plants on early stages of action stressors only, because low magnitudes of changes in RGB indices, the NDVI, PRI, and Fv/Fm (Figure 1, Figure 2, Figure 3, Figure 4, Figure 5 and Figure 6) decreased efficiency of the analysis under these conditions.

Most of the RGB indices excluding b were strongly correlated with Fv/Fm, NDVI and PRI; absolute values of the Pearson correlation coefficients were lower in wheat plants under the salinization action. This result was in accordance with intricated dynamics of changes in RGB indices in this variant. It should be noted that ExG and VARI seemed to be strongly correlated with Fv/Fm, the NDVI, and PRI in different experimental variants (pea/wheat, drought/salinization); these indices were selected as the most effective to estimate Fv/Fm, the NDVI, and PRI.

**Figure 7:**
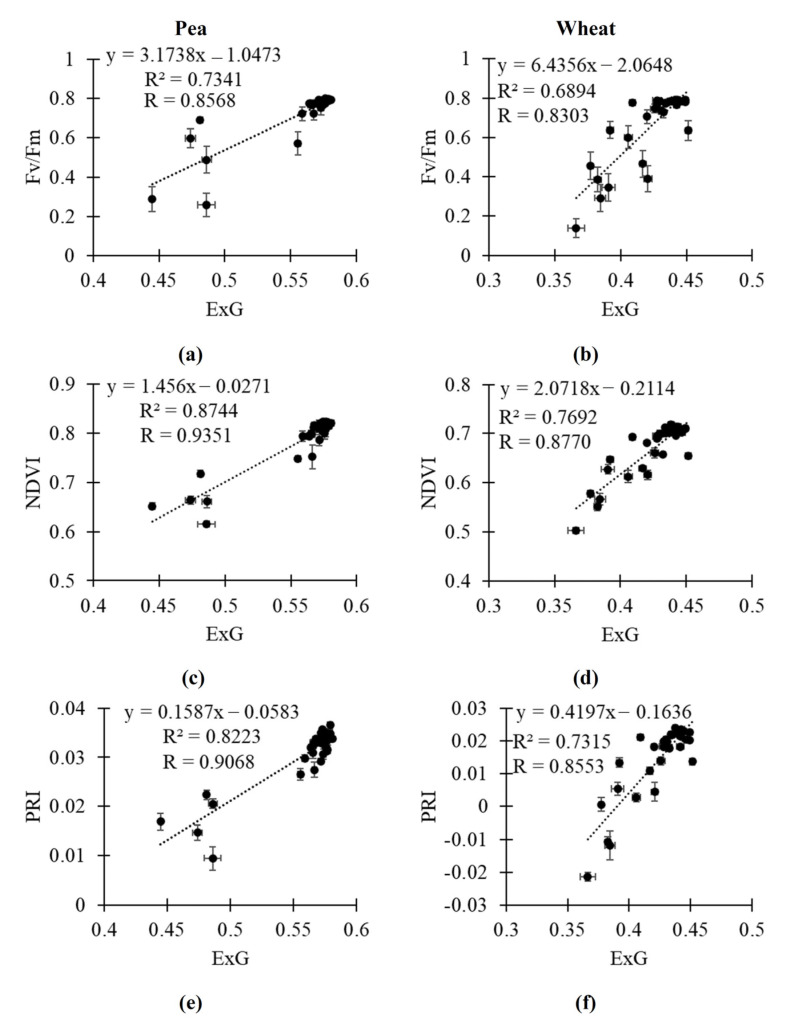
Relationships of ExG to Fv/Fm, NDVI, and PRI. (**a**) Scatter plots between ExG and Fv/Fm in pea plants. (**b**) Scatter plots between ExG and Fv/Fm in wheat plants. (**c**) Scatter plots between ExG and NDVI in pea plants. (**d**) Scatter plots between ExG and NDVI in wheat plants. (**e**) Scatter plots between ExG and PRI in pea plants. (**f**) Scatter plots between ExG and PRI in wheat plants. Average values of parameters in control plants, plants under soil drought, and plants under salinization in all time points were analyzed (*n* = 30). R^2^ and R are determination and correlation coefficients, respectively.

**Figure 8:**
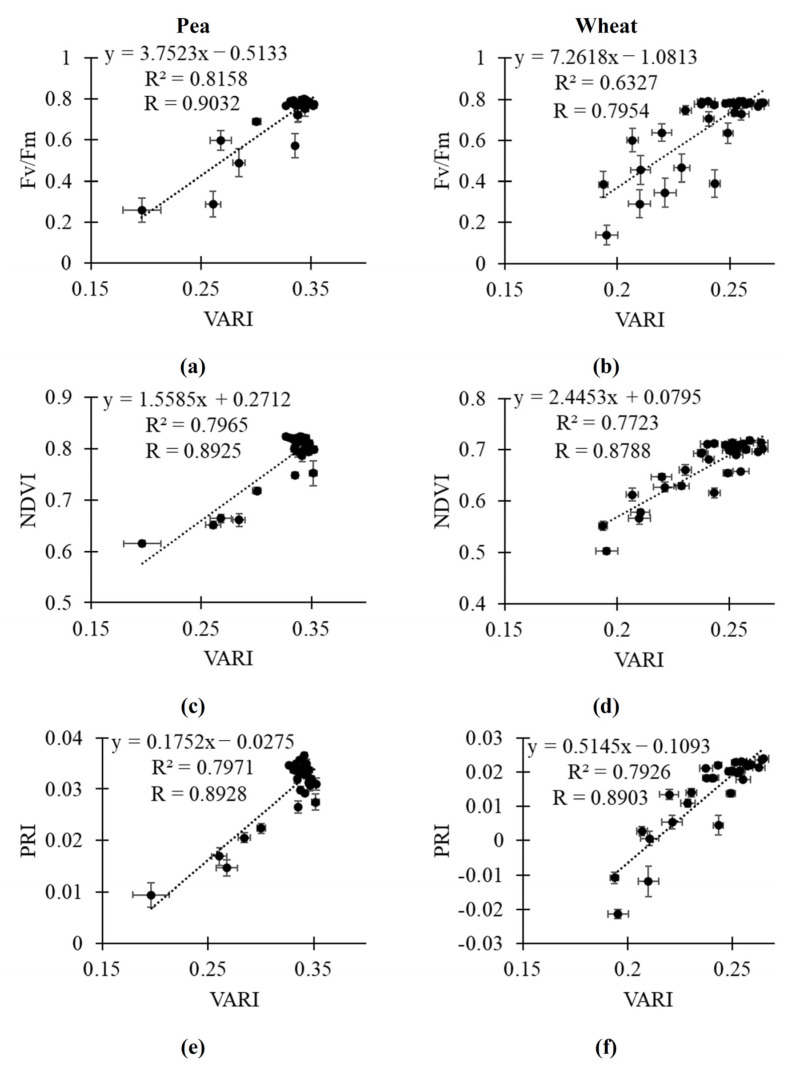
Relationships of VARI to Fv/Fm, NDVI, and PRI. (**a**) Scatter plots between VARI and Fv/Fm in pea plants. (**b**) Scatter plots between VARI and Fv/Fm in wheat plants. (**c**) Scatter plots between VARI and NDVI in pea plants. (**d**) Scatter plots between VARI and NDVI in wheat plants. (**e**) Scatter plots between VARI and PRI in pea plants. (**f**) Scatter plots between VARI and PRI in wheat plants. Average values of parameters in control plants, plants under soil drought, and plants under salinization in all time points were analyzed (*n* = 30). R^2^ and R are determination and correlation coefficients, respectively.

Finally, we analyzed scatter plots between the investigated RGB indices and Fv/Fm, NDVI and PRI to provide linear regression which could be used to quantitatively estimate Fv/Fm, the NDVI, and PRI on the basis of RGB indices (Figure 7, Figure 8 and Figures S1–S4). Average values of parameters in control plants and plants under drought and salinization in all time points (the 1st, 3rd, 5th, 8th, 10th, and 12th days) were used in these scatter plots. It was shown that the linear regressions could quantitively describe Fv/Fm, the NDVI, and PRI on the basis of ExG (Figure 7) and VARI (Figure 8); R^2^ was more than 0.7 in all variants excluding estimation of Fv/Fm in wheat plants. Linear regressions on basis of r (Figure S1) and VEG (Figure S4) had lower determination coefficients; however, these regressions can also be effective to estimate Fv/Fm, the NDVI, and PRI. Estimation of Fv/Fm, the NDVI and PRI on the basis of b was not effective (Figure S3). It should be additionally noted that linear regressions describing relationships of g to Fv/Fm, NDVI and PRI had R^2^ > 0.70 in all investigated variants (Figure S2).

Thus, our results showed that ExG, VARI, and g could be effective tools to quantitively estimate the investigated narrow-band reflectance indices (NDVI and PRI) and the potential quantum yield of photosystem II (Fv/Fm).

## 3. Discussion

The global climatic changes and faulty irrigations increase areas which are subject to influence of salinization and drought [1,2]. Both factors can induce stress changes in plants [1,2,3,4,5,52] including inhibition of photosynthesis and decrease in productivity. Considering the influence of soil drought and salinization on plant cultivation, the development of methods of early and low-cost remote and proximal sensing of action of these stressors on plants is an important applied task [12,13,16,31].

Multispectral and hyperspectral imaging, which is the basis of calculation of narrow-band reflectance indices, is a powerful tool of this sensing [7,12,20]. In particular, the NDVI [22], which is sensitive to changes in photosynthetic biomass [22] and chlorophyll content in leaves [24,25], and the PRI [23], which is sensitive to acidification of lumen [16,29,30] (and, therefore, to photosynthetic activity [53]) and to changes in content of photosynthetic pigments [54], are widely used indicators of plant health [12]. However, multispectral and hyperspectral imaging requires sophisticated measuring systems that restricts its using. In the current work, we investigate alternative RGB imaging, which is based on using simple color cameras [13,55], and analyze the sensitivity of some RGB indices (r, g, b, ExG, VEG, and VARI) to the action of soil drought and salinization and their efficiency to estimate key narrow-band reflectance indices (NDVI and PRI) and the potential quantum yield of photosystem II.

Our results show that both soil drought and salinization influence most of the investigated RGB indices (Figure 2, Figure 3, Figure 5 and Figure 6). In particular, g, ExG, VEG, and VARI are decreased under the action of drought and salinization; in contrast, r is increased under this action. These changes can be initiated on the early stage of drought and salinization action on plants; e.g., changes in g and ExG can be initiated in the 1st (pea plants, 400 mM NaCl) or 3rd (pea and wheat plants, drought) days of action of these stressors. In contrast, changes in Fv/Fm and the NDVI are observed in the 8th and 5th days, respectively (in pea and wheat plants under drought; in pea plants under the 400 mM NaCl treatment); it means that RGB indices can detect stress changes in plants more effectively than Fv/Fm and the NDVI. Potentially, the PRI can have similar sensitivity to action of stressors (e.g., changes in the PRI are induced in the 1st day in wheat under soil drought). However, the time of initiation of changes in PRI is strongly varied: from the 1st day (Figure 1f) to the 8th day (Figure 1e); this variability can decrease efficiency of the PRI to estimate the action of drought and salinization.

It should be noted that changes in b are differed from changes in the other investigated RGB indices; particularly, b is weakly sensitive to the drought action (Figure 2c and Figure 3c), but it is strongly sensitive to the NaCl treatment (Figure 5c and Figure 6c) including even the 100 mM NaCl concentration. We suppose that this result is caused by strong dependence of b on reflectance in the blue spectral region (i.e., on B). It is known [56] that the normalized red–green index, which is not dependent on B, is strongly sensitive to the drought action; in contrast, normalized red–blue index and normalized green–blue index, which are dependent on B, are weakly sensitive to drought.

The different sensitivity of b, which is mainly based on B, and other investigated RGB indices, which are mainly related to R (r), G (g), or all R, G, and B (ExG, VEG, and VARI), can be caused by different mechanisms of changes in reflectance in different spectral bands. It is known that chlorophylls absorb light in red and blue spectral regions and carotenoids absorb light in blue, and less so in green regions [8,23,57]. The degradation of chlorophylls and carotenoids induces increasing reflectance; the fast degradation of chlorophylls in comparison to carotenoids contributes to additional increasing reflectance in the red spectral band [14,58,59,60,61]. In contrast, the degradation of chlorophylls and carotenoids with similar rates, which is shown in plants under the action of salinization or drought in some works [61,62,63], can additionally increase reflectance in the blue spectral region because both chlorophylls and carotenoids influence this reflectance. Thus, high sensitivity of b to the NaCl treatment and low sensitivity to the drought action can be potentially related to different changes in contents of chlorophylls and carotenoids; maybe, these differences can be related to specific influence of salinization (e.g., through accumulation of Na^+^).

In contrast, the water stress, which is induced by both drought and salinization, influences red and green reflectance [64,65] changing, e.g., r, g, VEG, and VARI [64,66,67]. These changes can be correlated with water content [66,67]; however, their correlations with the content of chlorophylls [67] support the participation of changes in concentrations of pigments in these responses.

Thus, investigated RGB indices are sensitive to the action of drought and salinization. Times of initiation changes in RGB indices (r, g, ExG, VEG, and VARI for drought and salinization; b for salinization) and in narrow-band reflectance indices (NDVI and PRI) are varied in different experimental variants; earlier changes in RGB indices can be observed. As a whole, both RGB indices and narrow-band reflectance indices have approximately similar sensitivity to early changes in wheat and pea plants under action of stressors. Stability of dynamics of RGB indices under control conditions are lower than this stability of the PRI and, especially, the NDVI; however, fluctuations in control dynamics are not strongly prevent revealing significant changes in RGB indices. Changes in RGB indices and narrow-band reflectance indices are large and stable under action of strong drought (from the 8th day) and salinization (400 mM from the 10th day); changes in these indices under action of moderate stressors (drought before the 8th day, 400 mM NaCl before the 10th day, 100 and 200 mM NaCl) can be low or instable.

Finally, our results show that the investigated RGB indices (excluding b) are strongly related to key narrow-band reflectance indices (NDVI and PRI) and to Fv/Fm (Table 1). Figure 7, Figure 8, Figure S1, Figure S3, and Figure S4 show regression equations which can be used to quantitatively estimate the NDVI, PRI, and Fv/Fm on basis of RGB indices. Relationships of the investigated RGB indices to the NDVI and PRI are in good accordance with the few works, showing correlations between RGB indices and narrow-band reflectance indices (see, e.g., [41]). Additionally, the relationships are in good accordance with works [42,43,44] showing reconstruction of reflectance spectra or reflectance indices on the basis of RGB imaging with using machine learning. Thus, our results show that some RGB indices (particularly, ExG, VARI, and, probably, g) can be used to estimate the NDVI and PRI; i.e., RGB imaging can be used instead of multispectral or hyperspectral imaging in this case.

It is known that calculation of the NDVI is based on two narrow reflectance bands in red and near-infrared spectral regions [12,22] and calculation of the PRI is based on two narrow reflectance bands in green and yellow spectral regions [16,23]. It means that both the NDVI and PRI can be related to RGB indices, which are dependent on red and green spectral regions. ExG and VARI are calculated on the basis of difference between reflectance in these spectral regions [13]; it can explain strong correlation of these RGB indices to the NDVI and PRI.

Relationships of the most of the investigated RGB indices to Fv/Fm show that these indices can be used to estimate photosynthetic parameters under the action of stressors (drought and salinization). It means that simple color cameras can be potentially used instead of PAM fluorimeters (at least, to approximately estimate Fv/Fm on basis of regressions adapted to specific plant species). It should be noted that changes in investigated RGB indices, which are based on reflectance in broad spectral regions [13], and Fv/Fm, which is based on intensities of fluorescence of photosystem II under measuring and saturation light [45], can be indirect. Considering similar decreasing content of photosynthetic pigments and Fv/Fm under the strong water stress [46,47], we suppose that the decreasing content of these pigments under drought and salinization is the basis of relationships between RGB indices, which can be strongly dependent on concentrations of the pigments [13,67], and Fv/Fm, which is decreased at damage of photosynthetic machinery under the action of stressors [45,46,47,48,49,50,51].

Thus, our results show that (i) the investigated RGB indices (r, g, b, ExG, VEG, and VARI) can be sensitive to action of soil drought and salinization in pea and wheat plants; (ii) r, g, ExG, VEG, and VARI are strongly related to the NDVI and PRI; (iii) r, g, ExG, VEG, and VARI are strongly related to Fv/Fm. These results show that investigated RGB indices can be used to develop methods of early detection of action of stressors on plants and quantity estimation of the NDVI, PRI, and Fv/Fm on the basis of images measured by color cameras. These methods will be in demand by farmers and agronomists to provide precision plant cultivation and by environmental scientists to provide ecological monitoring. There are following potential advantages of using of RGB indices: (i) low cost of color cameras which provides their availability; (ii) low weight of color cameras which provides their localization on various mobile platforms (e.g., unmanned aerial vehicles); (iii) availability of numerous equipment with built-in color cameras (e.g., smartphones). Potentially, these advantages can provide wider application of methods of estimation of the NDVI, PRI, and Fv/Fm on the basis of RGB imaging in comparison with direct estimation of these parameters by hyperspectral and multispectral cameras or PAM fluorometers.

However, our results have some limitations requiring future investigations. First, plants were investigated in the measuring stand under controlled laboratory conditions. It means that estimation of efficiency of these RGB indices under open-air conditions and sunlight is an important task of future investigations. Second, the sensitivity of investigated RGB indices to the action of other stressors (e.g., non-optimal temperatures) requires future investigations. Third, our results show that coefficients of linear regression equations are differed for wheat and pea plants. It means that the monitoring of other plant species can require additional parameterization of the regression equations. Fourth, developing methods of quantity estimation of other narrow-band reflectance indices and photosynthetic parameters is also an important task of future investigations.

Finally, the analysis of ways of additional increasing efficiency of estimation of the NDVI, PRI, and Fv/Fm remains an important task of future investigations. Potentially, this increasing can be based on an analysis of optical models of plant leaf or on investigation of spatial heterogeneity of RGB indices (at least, we previously showed that spatial heterogeneity in reflectance at 530 nm is effective estimator of Fv/Fm under salinization [68]). Works, which show the efficiency of using texture analysis of color images to estimate characteristics of plants [69,70] additionally support an importance of analysis of spatial distribution of RGB characteristics. Using machine learning, which can include using random forest, support vector machine, extreme gradient boosting, and other methods [71], is another way of increasing efficiency of remote and proximal sensing of plants based on their color images [13].

## 4. Materials and Methods

### 4.1. Cultivation of Wheat and Pea Plants and Induction of Soil Drought and Salinization

Two–four-week-old pea (*Pisum sativum* L., cultivar “Albumen”) and wheat (*Triticum aestivum* L., cultivar “Daria”) plants were used in the current investigation in accordance with [56]. Plants were cultivated under controlled conditions of the vegetation room at 23 °C and 16 h photoperiod; humidity was not controlled. The illumination was provided by luminescent lamps FSL YZ18RR (Foshan Electrical And Lighting Co., Ltd., Foshan, China). Growth light intensity was about 50 µmol m^−2^s^−1^. The Thorlabs PM100D optical power meter (Thorlabs Inc., Newton, MA, USA) with an S120VC sensor (200–1100 nm) was used to measure intensity of illumination.

Plants were cultivated in pots with the peat soil “Morris Green” (Pelgorskoe M, Ryabovo, Russia); each pot contained 9 plants. Pots with plants were placed in pallets (4 pots per pallet for pea plants and 15 pots per pallet for wheat plants), which were used for imaging. As a result, 36 pea plants and 135 wheat plants were investigated in each experimental variant.

Irrigation by 50 mL of water was carried out three times per week (Monday, Wednesday, and Friday); the water regime was not changed for control plants. In accordance with [56], soil drought and salinization were initiated after 2 weeks of plant cultivation. Soil drought was initiated by the absence of irrigation: the 1st day of drought corresponded to the 3rd day after termination of irrigation. Salinization was initiated by irrigations with 50 mL of 100, 200, or 400 mM NaCl solution three times per week.

The total duration of action of both stressors was 12 days in accordance with our previous results [56]. The final relative water content (RWC) in shoots of plants was calculated on the basis of fresh and dry weights. Shoots of the plants were dried at 100 °C for 4 h using a thermostat TV-20-PZ-K (Kasimov Instrument Plant, Kasimov, Russia). It was shown (Figure S5) that the final RWC was significantly decreased after drought (from 90% to 20–30%). Salinization significantly decreased RWC in most variants; particularly, the final RWC after 12 days of salinization was about 40–60% at 400 mM NaCl. These results showed that soil drought and salinization induced water stress in wheat and pea plants and could be used in the further analysis.

### 4.2. Measurement of Potential Quantum Yield of Photosystem II in Plants

Before measurements, plants were adapted for at least 60 min under dark conditions. The potential quantum yield of photosystem II (Fv/Fm) was measured by the handheld pulse amplitude modulation (PAM) fluorometer FluorPen FP 110-LM/D (Photon Systems Instruments, Drasov, Czech Republic). The wavelength of light from LED emitter was about 455 nm, the wavelength range of detector was from 667 to 750 nm. Intensity of saturating pulse light was 2400 μmol m^−2^ s^−1^, and intensity of measuring pulse light was 0.027 μmol m^−2^ s^−1^. Fv/Fm was calculated using Equation (1) [72]:

(1) $$Fv/Fm=\frac{Fm-F_{0}}{Fm}$$

where Fm and F_0_ are maximal and dark fluorescence yields of photosystem II, respectively.

### 4.3. Measurement of Narrow-Band Reflectance Indices

The hyperspectral images of pallets with plants were measured by the hyperspectral camera Specim IQ (Specim, Spectral Imaging Ltd., Oulu, Finland) in accordance with [56]. Specim IQ had 400–1000 nm spectral range, 204 spectral bands, approximately 3 nm sampling interval, and 0.2-megapixel matrix.

The camera was standardly fixed in the measuring stand above pallet with plants, the distance between plants and camera was about 1 m. Plants were illuminated by halogen lamps during measurements; the light intensity was about 200 µmol m^−2^s^−1^. The white reflectance standard supplied with the camera Specim IQ was used for each measurement.

We calculated two narrow-band reflectance indices, the NDVI [22] and PRI [23] using Equations (2) and (3):

(2) $$NDVI=\frac{R_{780}-R_{680}}{R_{780}+R_{680}}$$

(3) $$PRI=\frac{R_{531}-R_{570}}{R_{531}+R_{570}}$$

where R_780_ and R_680_ are reflectance of plants at 780 and 680 nm, respectively; R_531_ and R_570_ are reflectance at 531 and 570 nm, respectively.

**Figure 9:**
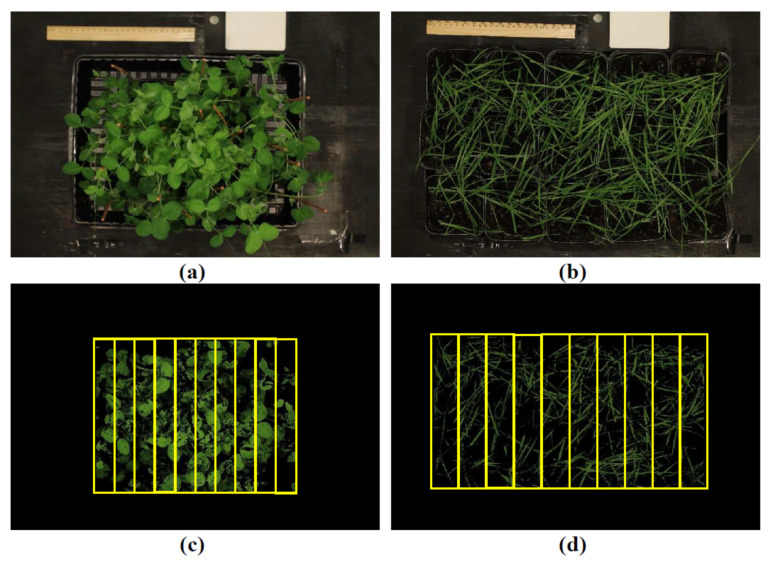
Examples of RGB images of pea (**a**) and wheat (**b**) plants before analysis. These images of pea (**c**) and wheat (**d**) plants after excluding background pixels and including 10 rectangular regions of interest (ROIs), which are shown by yellow frames. The white reflectance standard is shown in upper right part of (**a**,**b**).

The “plant” pixels in images were identified with using threshold value of the NDVI: NDVI should be higher than 0.5 for pea and 0.4 for wheat in accordance with [56]; pixels with lower values of the NDVI were identified as background. Each image included 10 rectangular regions of interest (ROIs) in accordance with [56] (Figure 9). The values of narrow-band reflectance indices in “plant” pixels were averaged for each ROI.

Special program tools were developed to provide the described analysis. Programming language Python 3.8 with libraries spectral, numpy, scipy, and matplotlib was used.

### 4.4. Measurement of RGB Indices

Plants were photographed using the RGB camera Canon EOS 4000D (Canon Inc., Tokyo, Japan). The focus length was 35, ISO was 100, and mode of white balance was “tungsten” (automatic white balance was turned off). The camera and halogen lamps were fixed in the measuring stand, the distance between plants and camera was about 1 m. The light intensity was about 200 µmol m^−2^s^−1^. The white reflectance standard supplied with the camera Specim IQ was used for each measurement (Figure 9). R, G, and B were normalized on corresponded values, which were measured on the white standard.

Equations (4)–(9) [13,73,74,75] were used to calculate series of RGB indices including the normalized red coordinate (r), normalized green coordinate (g), normalized blue coordinate (b), excess green index (ExG), vegetative index (VEG), and visible atmospherically resistance index (VARI):

(4) $$r=\frac{R}{R+G+B}$$

(5) $$g=\frac{G}{R+G+B}$$

(6) $$b=\frac{B}{R+G+B}$$

(7) ExG=2·g−r−b

(8) $$VEG=\frac{G}{R^{a}B^{1-a}}\;a=0.667$$

(9) $$VARI=\frac{G-R}{G+R-B}$$

where R, G, and B were intensities of reflected light in red, green, and blue channels of RGB image, respectively. We used normalized R, G, and B in the current analysis to minimize influence of light source.

Equations (10) and (11) were used to identify “plant” pixels in RGB images.

(10) $${Mask}_{pea}=5\cdot 255\cdot \frac{0.1G}{0.1G+0.1R+B}$$

(11) $${Mask}_{wheat}=5\cdot 255\cdot \frac{0.1\left(G-R\right)}{0.1G+0.1R+B}$$

Mask_Pea_, which was more than 115.33 and less than 139.3, and Mask_Wheat_, which was more than 36.39 and less than 140, were used as criteria of “plant” pixels [49]. Other values of Mask_Pea_ and Mask_Wheat_ indicated “background” pixels. Each image included 10 rectangular ROIs in accordance with [56] (Figure 9). The values of RGB indices in “plant” pixels were averaged for each ROI.

Special program tools were developed to provide the described analysis. Programming language Python with libraries pillow, numpy, and scipy was used.

### 4.5. Statistics

Data were analyzed using descriptive statistics. Averages, standard errors, scatter plots, and quantities of repetitions are shown in figures. We used Student’s *t*-test for estimation of significance of difference between control and experimental plants. The relationships between parameters were analyzed using the Pearson correlation coefficients (R), which were calculated on basis of averaged values; these values were calculated for each experimental variant (control and drought or control and different NaCl treatments) and for each day of measurement. Averaged values were also used to form linear regressions for pea and wheat plants. Determination coefficients (R^2^) were used to estimate accuracy of the regressions. The Microsoft Excel 2016 was used to calculate statistical parameters.

## 5. Conclusions

Soil drought and salinization are key stressors decreasing growth and productivity of agricultural plants. As a result, the development of methods of low-cost remote and proximal sensing, which can be used to early reveal action of these stressors and to estimate plant characteristics, is an important applied task.

In the current work, we investigated the influence of soil drought and salinization on series of RGB indices (r, g, b, ExG, VEG, and VARI) and analyzed relationships of these indices to key narrow-band reflectance indices (NDVI and PRI) and to the potential quantum yield of photosystem II (Fv/Fm). It was shown that the investigated RGB indices (r, g, b, ExG, VEG, and VARI) were sensitive to the action of soil drought and salinization on pea and wheat plants and that r, g, ExG, VEG, and VARI were strongly related to the NDVI, PRI, and Fv/Fm.

Our results show that RGB imaging, which is based on measurements by simple color cameras, can be potentially used to quantitatively estimate important narrow-band reflectance indices (NDVI and PRI) and photosynthetic parameters (Fv/Fm) in plants under the action of stressors (at least, drought and salinization) without sophisticated measuring systems (hyperspectral and multispectral cameras and PAM fluorometers).

## Figures and Tables

**Table 1:** The Pearson correlation coefficients of RGB to Fv/Fm, NDVI, and PRI. Correlation coefficients were calculated on the basis of average values of parameters in all time points; average control and experimental values were separately analyzed (*n* = 12 for drought and *n* = 24 for salinization). Correlation coefficients with absolute value more than 0.58 were significant (*p* < 0.05).

**Pea under soil drought**			
	**Fv/Fm**	**NDVI**	**PRI**
r	−0.9891	−0.8983	−0.8883
g	0.7516	0.9388	0.9333
b	0.6250	0.2868	0.2781
ExG	0.7516	0.9388	0.9333
VEG	0.6303	0.8718	0.8682
VARI	0.9790	0.9434	0.9340
**Wheat under soil drought**			
r	−0.8620	−0.9291	−0.9361
g	0.9545	0.9813	0.9783
b	−0.8085	−0.7537	−0.7337
ExG	0.9545	0.9813	0.9783
VEG	0.9378	0.9763	0.9716
VARI	0.8842	0.9487	0.9506
**Pea under salinization**			
r	−0.8734	−0.7858	−0.7190
g	0.9602	0.9354	0.8980
b	−0.8038	−0.8685	−0.8802
ExG	0.9602	0.9354	0.8980
VEG	0.9619	0.9378	0.8987
VARI	0.9102	0.8231	0.7649
**Wheat under salinization**			
r	−0.7270	−0.7416	−0.7848
g	0.7504	0.8097	0.7746
b	−0.4328	−0.5073	−0.4143
ExG	0.7504	0.8097	0.7746
VEG	0.7562	0.8158	0.7798
VARI	0.7758	0.8096	0.8274

## Data Availability

Data are contained within the article and Supplementary Materials.

## Supplementary Materials

The following supporting information can be downloaded at https://www.mdpi.com/article/10.3390/plants14091284/s1. Figure S1: Relationships of r to Fv/Fm, NDVI, and PRI; Figure S2: Relationships of g to Fv/Fm, NDVI, and PRI; Figure S3: Relationships of b to Fv/Fm, NDVI, and PRI; Figure S4: Relationships of VEG to Fv/Fm, NDVI, and PRI; Figure S5: The relative water content in shoots of wheat and pea plants after 12 days of soil drought and salinization.
