# Supplementary material for: RGB Indices Can Be Used to Estimate NDVI, PRI, and Fv/Fm in Wheat and Pea Plants Under Soil Drought and Salinization
Source: Plants (Basel). 2025 Apr 23;14(9):1284. doi: 10.3390/plants14091284 (PMC12073610; doi:10.3390/plants14091284)
Supplement: Supplementary file 1 [file plants-14-01284-s001.zip › plants-3553880-supplementary.pdf]

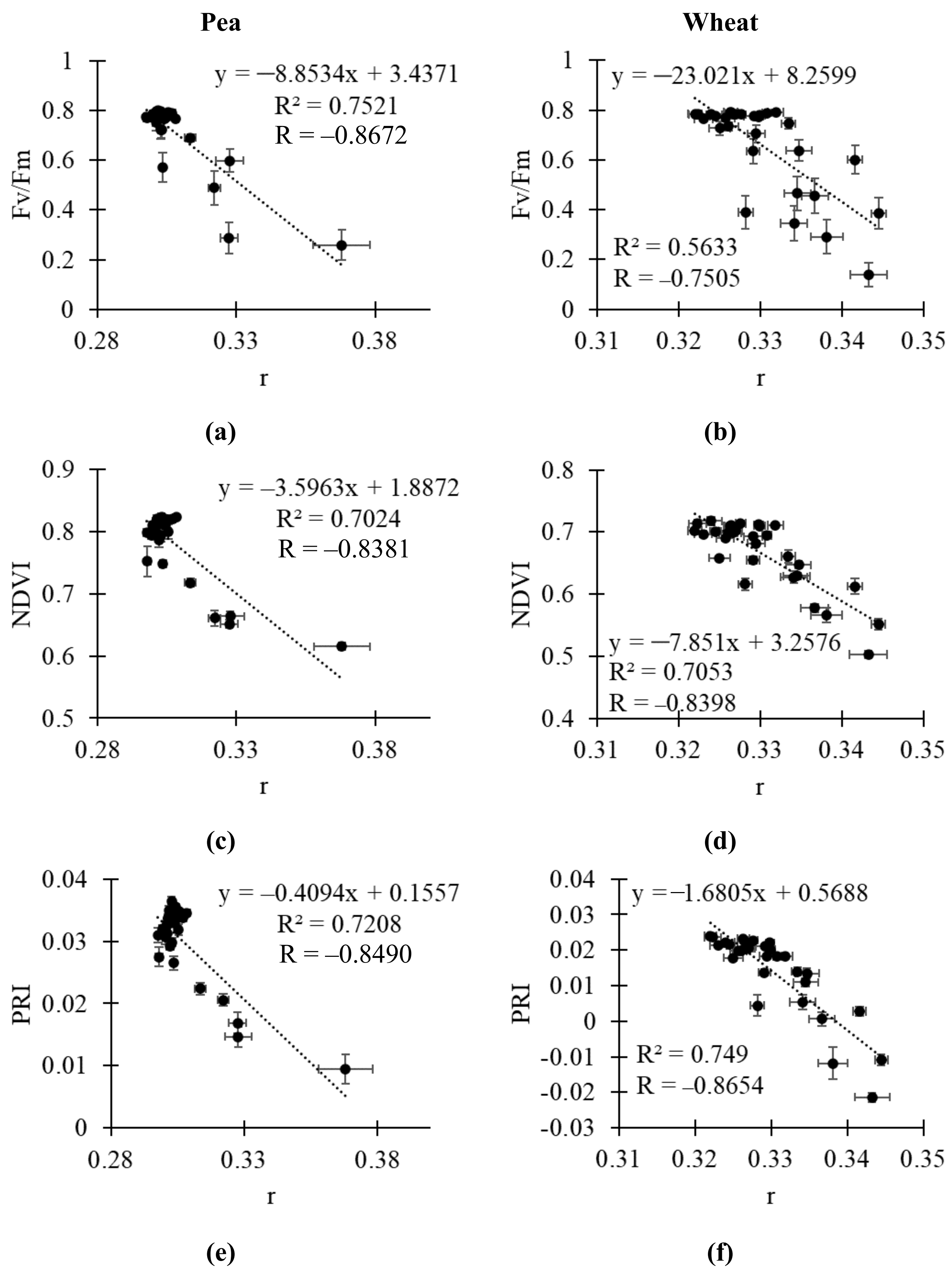

Figure S1. Relationships of r to Fv/Fm, NDVI, and PRI. **(a)** Scatter plots between r and Fv/Fm in pea plants. **(b)** Scatter plots between r and Fv/Fm in wheat plants. **(c)** Scatter plots between r and NDVI in pea plants. **(d)** Scatter plots between r and NDVI in wheat plants. **(e)** Scatter plots between r and PRI in pea plants. **(f)** Scatter plots between r and PRI in wheat plants. Average values of parameters in control plants, plants under soil drought, and plants under salinization in all time points were analyzed (n=18). R^2 and R are determination and correlation coefficients, respectively.

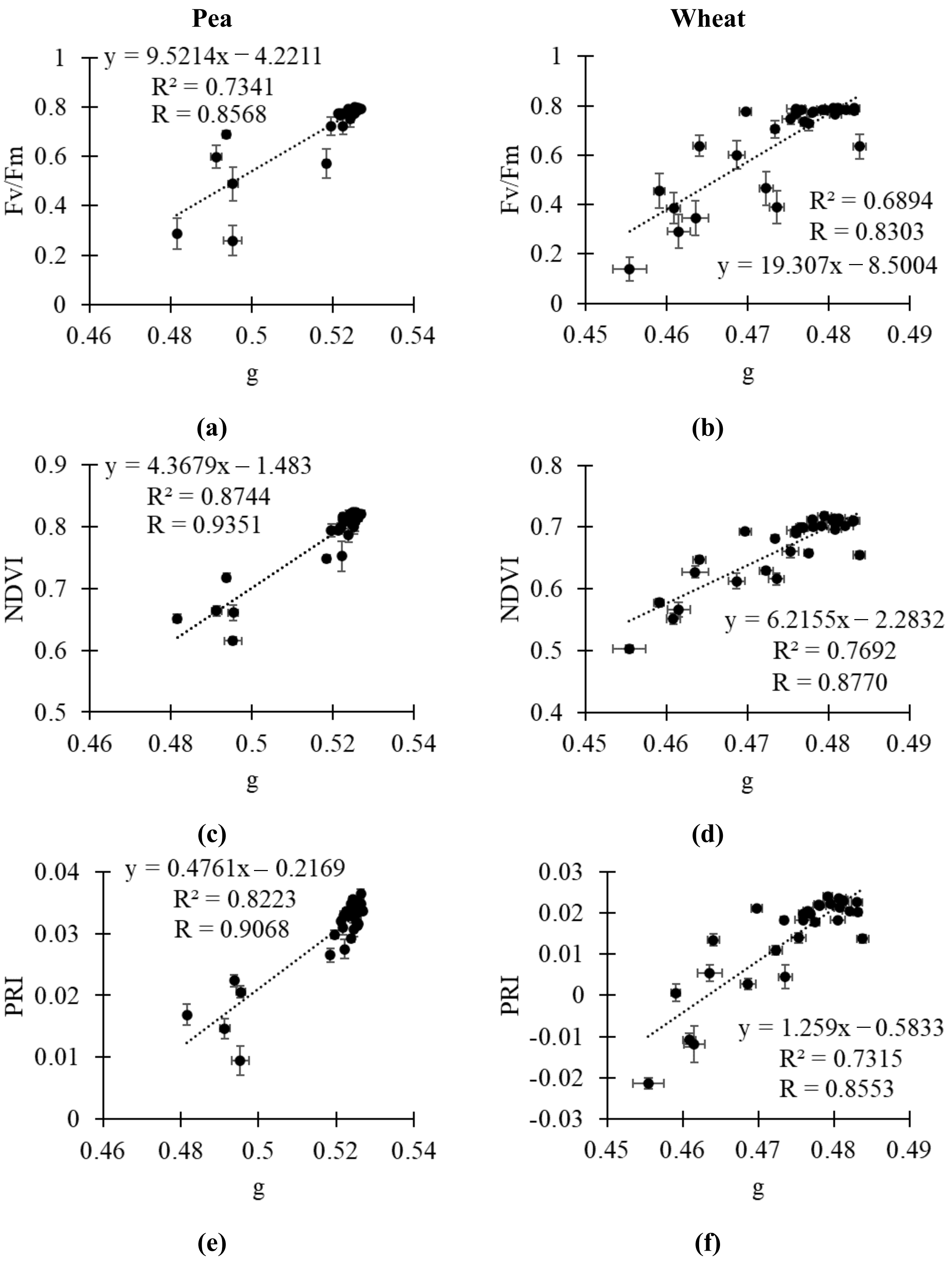

Figure S2. Relationships of g to Fv/Fm, NDVI, and PRI. **(a)** Scatter plots between g and Fv/Fm in pea plants. **(b)** Scatter plots between g and Fv/Fm in wheat plants. **(c)** Scatter plots between g and NDVI in pea plants. **(d)** Scatter plots between g and NDVI in wheat plants. **(e)** Scatter plots between g and PRI in pea plants. **(f)** Scatter plots between g and PRI in wheat plants. Average values of parameters in control plants, plants under soil drought, and plants under salinization in all time points were analyzed (n=18). R^2 and R are determination and correlation coefficients, respectively.

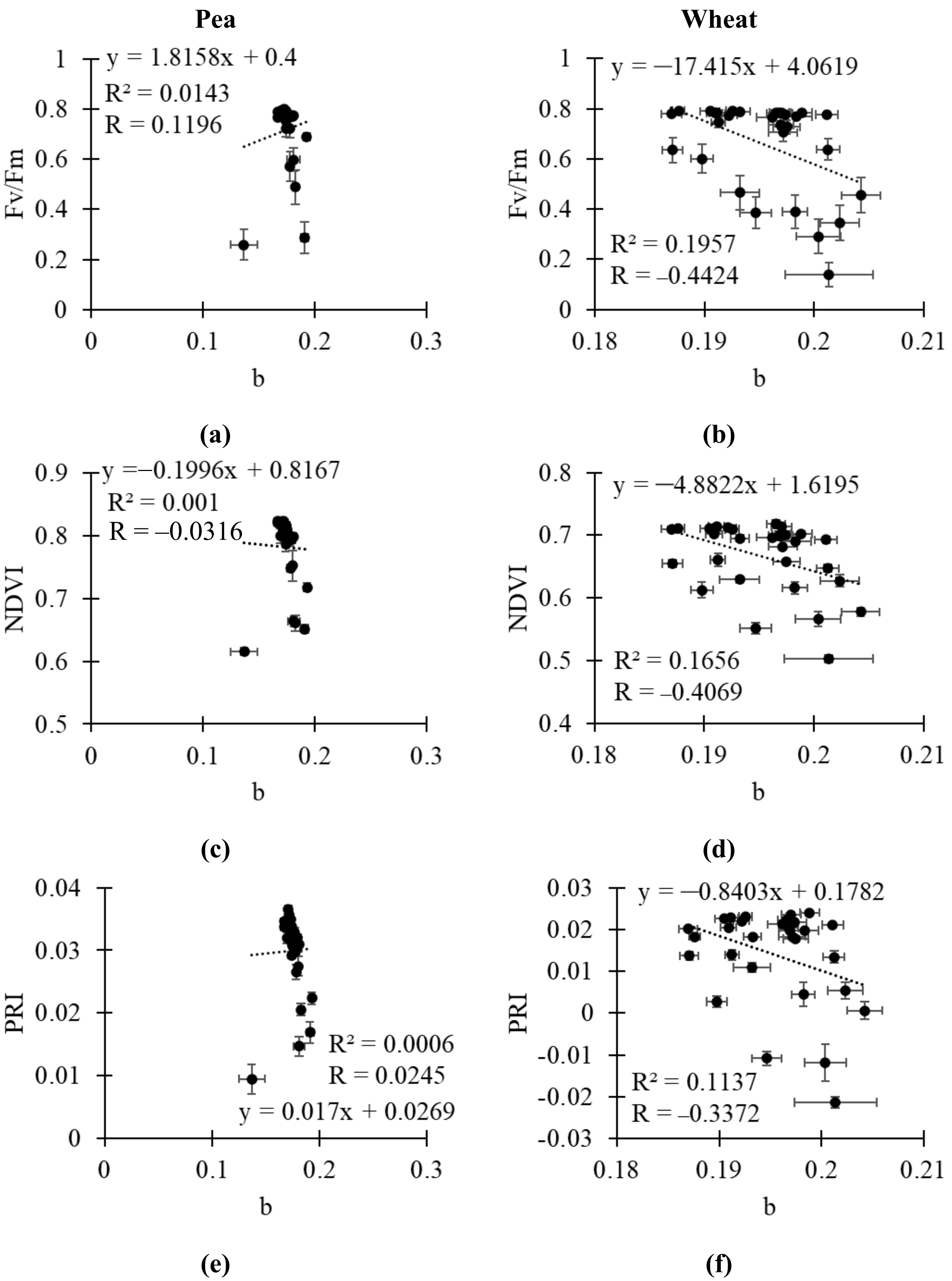

Figure S3. Relationships of b to Fv/Fm, NDVI, and PRI. **(a)** Scatter plots between b and Fv/Fm in pea plants. **(b)** Scatter plots between b and Fv/Fm in wheat plants. **(c)** Scatter plots between b and NDVI in pea plants. **(d)** Scatter plots between b and NDVI in wheat plants. **(e)** Scatter plots between b and PRI in pea plants. **(f)** Scatter plots between b and PRI in wheat plants. Average values of parameters in control plants, plants under soil drought, and plants under salinization in all time points were analyzed (*n*=18). R^2 and R are determination and correlation coefficients, respectively.

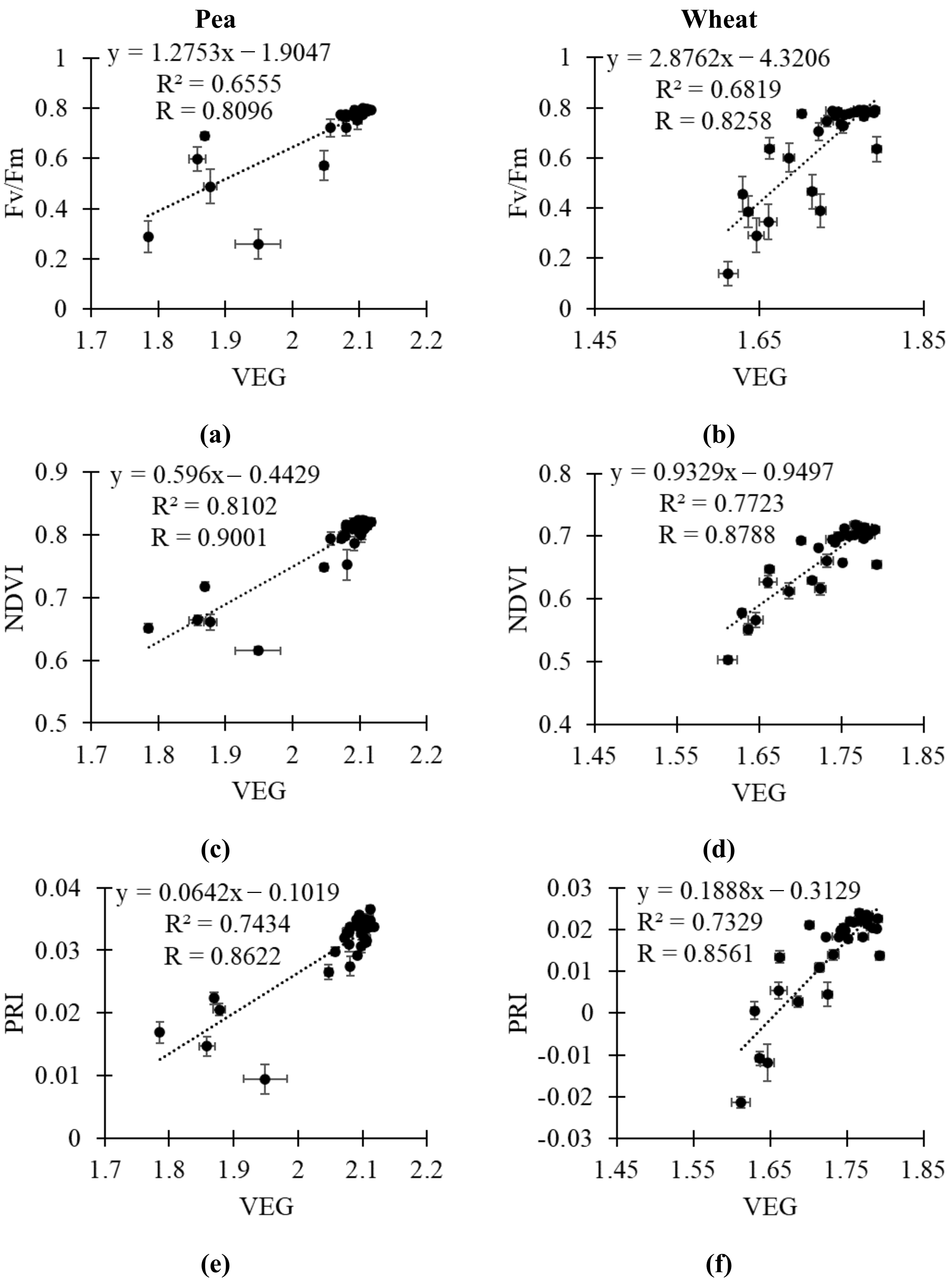

Figure S4. Relationships of VEG to Fv/Fm, NDVI, and PRI. **(a)** Scatter plots between VEG and Fv/Fm in pea plants. **(b)** Scatter plots between VEG and Fv/Fm in wheat plants. **(c)** Scatter plots between VEG and NDVI in pea plants. **(d)** Scatter plots between VEG and NDVI in wheat plants. **(e)** Scatter plots between VEG and PRI in pea plants. **(f)** Scatter plots between VEG and PRI in wheat plants. Average values of parameters in control plants, plants under soil drought, and plants under salinization in all time points were analyzed (n=18). R^2 and R are determination and correlation coefficients, respectively.

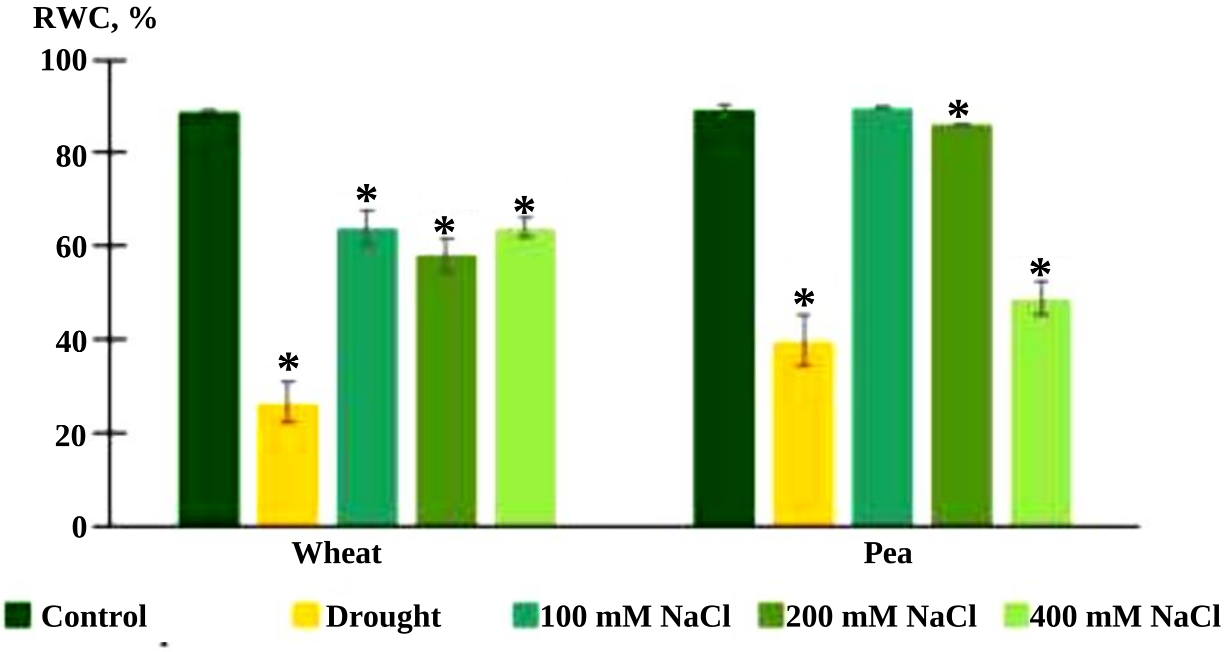

Figure S5. The relative water content (RWC) in shoots of wheat (n=15) and pea (n=5) plants after 12 days of soil drought and salinization. *, difference with control value was significant (p<0.05).
